# Retraction Note: Glycol chitosan incorporated retinoic acid chlorochalcone (RACC) nanoparticles in the treatment of Osteosarcoma

**DOI:** 10.1186/s12944-020-01416-2

**Published:** 2020-11-30

**Authors:** Yan-Guo Qin, Lan-Yu Zhu, Chen-Yu Wang, Bo-Yan Zhang, Qing-Yu Wang, Rui-Yan Li, Zhen Liu

**Affiliations:** 1 Department of Orthopedics, The Second Hospital of Jilin University, Changchun, 130041 Jilin China; 2 Nursing School, Changchun University of Chinese Medicine, Changchun, 130117 Jilin China; 3 Norman Bethune Medical School, Jilin University, Changchun, 130021 Jilin China

**Retraction to: Lipids Health Dis 14, 70 (2015)**

**https://doi.org/10.1186/s12944-015-0068-4**

The Editor in Chief has retracted this article [1] because of significant concerns regarding a number of Figures presented in this work, which question the integrity of the data. The authors have contacted the journal to say they were unable to replicate data presented in Figure 2A and C. In addition, concerns were raised by readers regarding:

- Figure 3A and 3B - similarities between bands within western blot
- Figure 4A- several areas appear to be repeated throughout the panels. It also appears Figure 4A was published by unrelated authors in a different article, which has since been retracted [2]
- Figure 6 - it appears the graphs representing results from different cell lines are very similar, but with different labels and quantification.
- Figure 7D - similarities between bands within each blot. It appears some bands have been repeated. It also appears a similar Western blot has been published earlier [3] in a different article by unrelated authors as Figure 4A.
- Figure 8 C, D, E and F where it appears several areas of the image are repeated throughout that image.

The authors did not respond to these concerns. They stated that the results cannot be reproduced.

The corresponding author Yan-Guo Qin stated on behalf of all co-authors that they agree to this retraction.
